# Prevention of hypertension due to long working hours and other work hazards is needed to reduce the risk of cardiovascular disease

**DOI:** 10.5271/sjweh.4196

**Published:** 2025-01-01

**Authors:** Paul Landsbergis, Mahee Gilbert-Ouimet, Xavier Trudel, Grace Sembajwe, Peter Schnall, Marnie Dobson, Devan Hawkins, Marc Fadel, Alexis Descatha, Jian Li

**Affiliations:** 1 SUNY Downstate School of Public Health, Brooklyn, NY, USA.; 2 Université du Québec, Rimouski, Canada.; 3 Université Laval, Quebec, Canada.; 4 Indiana University School of Public Health, Bloomington, IN, USA.; 5 University of California, Irvine, CA, USA.; 6 Massachusetts College of Pharmacy and Health Sciences, Boston, MA, USA.; 7 Univ Angers, CHU Angers, Univ Rennes, Inserm, EHESP, Irset (Institut de recherche en santé, environnement et travail) - UMR_S 1085, SFR ICAT, centre antipoison, Prevention, Angers, France.; 8 Epidemiology and Prevention, Donald and Barbara Zucker School of Medicine, Hofstra Univ Northwell Health, USA; 9 University of California, Los Angeles, CA, USA.

**Keywords:** blood pressure, heart disease, interventions work stressor

## Abstract

Hypertension is the foremost risk factor for cardiovascular disease (CVD), which is the leading cause of death globally. In some countries, such as the US, the prevalence of hypertension and working-age CVD mortality are increasing. CVD is also the most common work-related disease worldwide. Long working hours and other psychosocial stressors at work are important modifiable risk factors for hypertension and CVD. However, there has been inadequate attention paid to the primary prevention of work-related hypertension and CVD.

The state-of-the art method for blood pressure (BP) measurement is 24-hour ambulatory BP (ABP), necessary for accurate clinical decision making and to assess risk factors for BP elevation. Thus, ABP should be used in workplace screening and surveillance programs (along with surveys) to identify occupational risk factors, high-risk job titles, worksites and shifts, and evaluate programs designed to improve work organization.

For example, after 30 months of an organizational intervention designed to lower psychosocial stressors at work among >2000 public sector white-collar workers in Quebec, Canada, BP and prevalence of hypertension significantly decreased in the intervention group, with no change in the control group, and a significant difference between the intervention and control groups.

Further research is also needed on mechanisms linking work-related factors to hypertension and CVD, the cardiovascular effects of understudied work stressors, high-CVD risk worker groups, potential “upstream” intervention points, and country differences in working conditions, hypertension and CVD. Important organizational interventions, such as collective bargaining, worker cooperatives, or legislative and regulatory-level interventions, need to be evaluated.

Cardiovascular disease (CVD) is the leading cause of death globally (1). Hypertension, or high blood pressure (BP) – the foremost risk factor for CVD globally (2) – affects an estimated 1.28 billion adults aged 30–79 years worldwide, two-thirds of whom live in low- and middle-income countries (3).

Work-related CVD is the most common work-related disease worldwide (4). Long working hours (LWH) are a risk factor for heart disease (5), stroke (6) and recurrent coronary events (7). The World Health Organization and International Labor Organization estimated that 745 194 deaths and 23.3 million disability-adjusted life years from CVD were attributable to LWH in 2016 (8). However, inadequate attention has been paid to occupational risk factors for hypertension and CVD, particularly psychosocial stressors other than LWH and shift work [eg, job strain, effort reward imbalance (ERI)], and to work-based prevention, interventions and policies (9).

Recent commentaries in this journal have focused on working hours and chronic disease, including CVD (10). We hope to add to this discussion by focusing on the adverse effect of long working hours and other occupational risk factors on BP. We also discuss the validity of BP measurement, secular trends, cross-country differences, underlying mechanisms, and the workplace as a key location for identifying and preventing hypertension.

## Hypertension and CVD trends

Cross-cultural anthropological studies have consistently found that non-industrial societies, such as hunter-gatherers, have a very low prevalence of hypertension and that BP does not inevitably rise with age as in industrial societies (9). Recently, the age-adjusted prevalence of hypertension in the US increased for most gender-race-ethnicity groups between 2007–2010 and 2017–2020 (11). The prevalence of hypertension in the US is high among working-aged men (55% in age 45–54, 67% in age 55–64) and women (48.6% in age 45–54, 63.1% in age 55–64) (11). CVD mortality rates among working-age populations have also increased recently in some countries, especially in the US (see supplementary material, URL, section 3).

## The importance of where and when BP is measured

International guidelines “unanimously recommend … 24-hour ambulatory [ABP] monitoring as the state-of-the-art technique for BP measurement and as a prerequisite for individualizing hypertension management” (12). 24-hour ABP is superior to casually-measured/office BP (CBP) in predicting total and CVD mortality and overall and cause-specific CVD complications among hypertension patients and in population cohorts (12). “Masked” hypertension (elevated ABP but normal CBP), increases CVD risk nearly as much as sustained hypertension (12). Masked hypertension occurs in 10–15% of people with normal CBP, and masked uncontrolled hypertension occurs in 30–50% of treated hypertensive patients (13).

ABP is primarily used for diagnosis, treatment and medication adjustment. However, it needs to be far more extensively used in public health for prevention of hypertension. ABP can be used in workplace screening or surveillance programs (along with surveys) to identify high-risk job titles, worksites and shifts and evaluate the effectiveness of programs to improve work organization (14). Only ABP can reliably reveal BP variation over 24 hours, including during working hours, and the responsiveness of BP to work physical and psychosocial stressors (9, 12).

## LWH and ABP

A 1996 study found that the 24-hour average BP of Japanese male white-collar workers working overtime was higher than that of the control groups (15).

The adverse effect of LWH on ABP (measured during daytime working hours) and hypertension was documented in a 5-year prospective cohort of 3500 white-collar workers in Quebec City, Canada. Women working LWH (≥41 versus 35–40 hours/week) subsequently had higher diastolic ABP [+1.8 mm Hg (95% confidence interval (CI) 0.5–3.1)], and men working LWH had higher systolic [+2.5 mm Hg (95% CI 0.5–4.4) and diastolic (+2.3 mm Hg (95% CI 1.0–3.7)] ABP. ABP means were higher among workers exposed to both LWH and high family responsibilities, ie, number and age of children and extent of household chores and childcare tasks (16).

Participants in the Quebec study working 41–48 hours per week [prevalence ratio (PR)=1.54 (95% CI 1.09–2.19)] and those working ≥49 hours per week [PR=1.76 (95% CI 1.12–2.77)] also had a higher prevalence of masked hypertension. The prevalence of sustained hypertension (high BP based on both ABP and CBP) was also higher among those in the highest categories of working hours [PR_41–48_=1.33 (95% CI 0.99–1.76); PR_≥49_=1.66 (95% CI 1.15–2.50)] (17).

## Other psychosocial work stressors and ABP

ABP is associated with job strain, ERI (18) and other psychosocial work stressors (9), with effect sizes ranging from +1.9–11.0 mm Hg (systolic) and +1.5–7.0 mm Hg (diastolic) (18). ABP and prospective studies provide more consistent associations than studies of lower methodological quality, eg, studies of CBP (18). In other studies, masked hypertension has been associated with LWH, shiftwork and ERI (9, 17, 19). A number of potential mechanisms linking LWH, hypertension/ABP and CVD have been described (9).

## Hypertension as a mediator of the association of LWH and stroke

**Figure 1:**
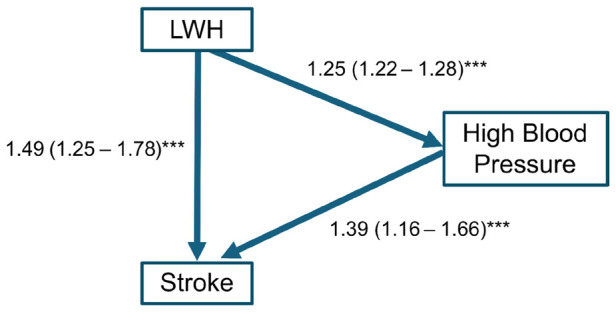
Odds ratio and 95% confidence interval of models of long work hours, hypertension and stroke, unpublished data from the French CONASTANCES cohort, ***P

In the French CONSTANCES cohort study (20), it was not feasible to measure BP every year, rather, researchers retrieved questionnaire data on history of diagnosed hypertension. In unpublished analyses of data from that cohort (see supplementary material section 1), the effect of exposure to ≥5 years of LWH (binary) on hypertension (binary) and stroke were both statistically significant, as well as the effect of hypertension on stroke (figure 1). This suggests hypertension is a direct and a mediating risk factor for stroke. In this cohort, approximately 18% (95% CI 12–33%) of the effect of LWH on stroke was mediated by hypertension (see supplementary material section 1).

## Intervention research

Until recently, only two small studies in Sweden examined the impact of interventions – changes in working conditions – on casual BP, with positive results for a flexible team-based work organization and for a set of interventions designed to reduce stressful working conditions in urban bus drivers (9). In addition, pooled data from four studies of work shift schedule changes showed a small significant reduction in casual systolic BP, but no significant change in casual diastolic BP (21)

A larger intervention study involving over 2000 white-collar workers at insurance services agencies in Quebec aimed to reduce the psychosocial work stressors of job strain and ERI through, for example, regular employee/manager or group meetings, organizational restructuring to reduce workload, slowing implementation of changes in work processes and computer software to allow adaptation, more flexible work hours, and career and skills development (22). At 30-month follow-up, ABP and hypertension significantly decreased in the intervention group, with no change in the control group. The differential decrease in ABP between the intervention and control groups was 2.0 mm Hg systolic (95% CI -3.0– -1.0), and 1.0 mm Hg diastolic (95% CI -1.7– -0.3), and hypertension prevalence decreased by 15%, PR=0.85, 95% CI 0.74–0.98 (14).

## A proposed action research agenda

Based on the research presented earlier, we propose an action research agenda comprising workplace surveillance, etiological research, a range of programs to improve working conditions, and intervention research.

*Conduct workplace screening and surveillance.* Relying solely on CBP leads to errors in hypertension diagnosis and treatment, underestimating the strength of associations between working conditions and BP, as well as between intervention status and BP. The development of valid and reliable wearable ABP monitors (23) allow for greater use of ABP in worksite screening, surveillance and intervention studies, together with other measures of health (eg, sleep), physical activity, and the physical and psychosocial work environment.

*Assess etiology. Hypertension* (ideally measured by ABP) is an important modifiable mediator of the association between long working hours (and other work stressors) and CVD. Adjusting for hypertension in studies of work and CVD could be overadjustment, thus, more mediation analyses are needed. More knowledge is needed on the *cardiovascular effects of understudied work stressors*, such as threat avoidant vigilance (eg, urban bus driving), organizational or workplace injustice (procedural injustice and distributive injustice), workplace discrimination, harassment and violence (including harassment and bullying), job insecurity, electronic performance monitoring, emotional demands/labor, non-standard work arrangements and digital platforms. Investigation is needed into the *specific modifiable working conditions among vulnerable worker populations at high risk of CVD*, such as professional drivers, low-wage, manual and older workers, workers with pre-existing CVD or exposed to psychosocial stressors combined with high occupational physical activity or environmental heat (eg, retail, warehouse, farm, and construction workers) (9). Greater understanding is needed of the *specific causes of LWH and other work stressors* to ascertain potential “upstream” or distal intervention points, including informal (versus formal) employment, privatization of public services, deregulation, inadequate public sector budgets, precarious employment, social inequalities, lean production and new public management (9). Further assessments should be carried out of the *differences between countries in working conditions and health*, which may be influenced by union density, psychosocial safety climate and legislation (see supplementary material section 4). To what extent may such factors help to explain worsening health status (including CVD and hypertension) in the US compared to peer countries?

*Conduct interventions and prevention programs, enact regulations/guidelines.* More studies are needed comparing the *costs and benefits* of hypertension medication and CVD treatment with those of work-related interventions aimed at preventing hypertension and CVD. National *laws, regulations or guidelines* on LWH or other work organization or psychosocial stressors need to promote ABP monitoring as a key surveillance method. Important *organizational interventions*, such as collective bargaining, worker cooperatives, or legislative and regulatory-level interventions, are rarely evaluated or included in intervention review articles. US examples include laws providing for better nurse-patient staffing ratios, bans on mandatory overtime, paid sick days, paid family leave or retail worker schedule predictability (9). In Quebec, Canada, a law modernizing the occupational health and safety system will oblige all employers to measure and analyze psychosocial risks at work (prioritizing workplace violence) starting in 2025 (see supplemental material section 4). Such interventions need to be evaluated for their impacts on BP and CVD.

## Supplementary material

Supplementary material
