# Supplementary material for: Prevention of hypertension due to long working hours and other work hazards is needed to reduce the risk of cardiovascular disease
Source: Scand J Work Environ Health. 2024 Dec 30;51(1):48–52. doi: 10.5271/sjweh.4196 (PMC11697614; doi:10.5271/sjweh.4196)
Supplement: Supplementary material [file SJWEH-51-48-S001.pdf]

Prevention of hypertension due to long working hours and other work hazards is needed to reduce the risk of cardiovascular disease[1]

by Paul Landsbergis, PhD,[2] *Mahee Gilbert-Ouimet, PhD, Xavier Trudel, PhD, Grace Sembajwe, ScD, Peter Schnall, MD, Marnie Dobson, PhD, Devan Hawkins, ScD, Marc Fadel, MD, Alexis Descatha, MD, Jian Li, MD*

1. Supplementary material
2. Correspondence to: Paul Landsbergis, School of Public Health, State University of New York (SUNY)-Downstate Health Sciences University, 450 Clarkson Ave., Brooklyn, NY 11203, USA. [E-mail: paul.landsbergis@downstate.edu]

I. BP as a mediator of the association of long work hours and stroke

The recent editorial by Härmä et al. on the effects of long work hours (LWH) on health called for a better understanding of underlying mechanisms (1). Recent reviews found that exposure to LWH increased risk of heart disease (2) and stroke (3). Given the major role of high blood pressure (hypertension) as a cardiovascular disease (CVD) risk factor, the potential mechanism of high BP as a mediator between LWH and stroke is of interest.

Baron and Kenny's (4) method was used in an unpublished analysis of data from the French CONSTANCES cohort to investigate mediated pathways (see (5, 18) for the study design). It was not feasible to measure ABP in this study, rather, researchers used an algorithm to identify participants with hypertension based on questionnaire data on history of diagnosed hypertension, BP measured during a physical exam (>140 systolic BP or >90 diastolic BP) and hypertension medication taken six months before inclusion. The effect of LWH (binary, exposed to 5 years or more of LWH) on diagnosed hypertension (binary), and on stroke were both statistically significant, as well as the effect of hypertension on stroke (Figure 1). This suggests the role of hypertension as a direct risk factor for stroke, but also as a mediating factor, with approximately

18% (95% confidence interval 12-33%) of the effect of LWH on stroke mediated by hypertension.

Figure 1. Odds ratio and 95% confidence interval of models for long work hours (LWH) and hypertension (high blood pressure), unpublished data from the CONSTANCES cohort (5), using the Baron and Kenny method, ***p<.001 (4).

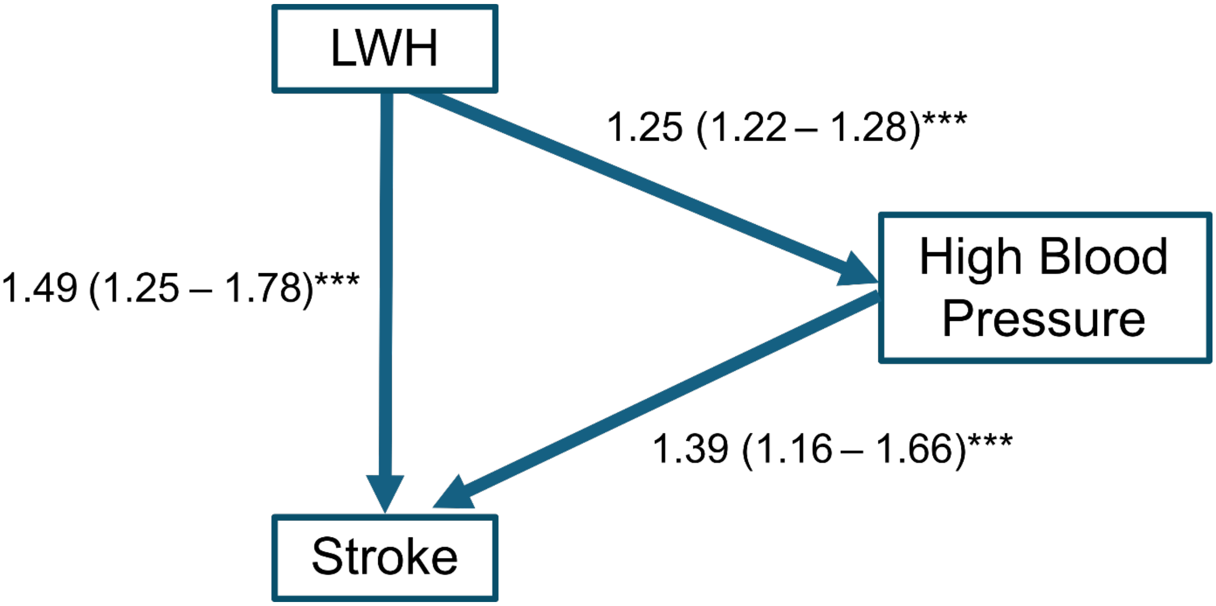

2. Wearable monitors for ABP and other health measures (e.g., physical activity, sleep) for clinical purposes and for workplace surveillance and interventions

In recent years, affordable wearable devices that track physical activity, sleep, and other vital signs, such as BP, have been integrated into research, and these wearables should be validated for intervention studies. The prospect of monitoring ABP and treating ABP measures as validated exposure metrics, also for clinical purposes, is an attractive one. The use of ABP as a signal for circadian rhythm disruptions, or acute stress alerts, for example, could lead to interventions that reduce or prevent any long-term damage to the cardiovascular system. In early iterations of the Work-Family health study, daily diary entries revealed that workplace stressors

were directly associated with spikes in cardiometabolic risk factors. This work led to a series of randomized workplace interventions that also highlight a need for ABP integration to better understand pathways of workplace stress to CVD outcomes and ways in which to intervene early (6).

3. Worsening cardiovascular and working conditions trends, especially in the U.S.

The rate of decline in CVD mortality slowed considerably in most developed countries in recent years, particularly in working age groups, and CVD death rates rose in seven countries for at least one sex in 2017 (7). In the U.S., between 2020 and 2022, there were 9% more U.S. CVD deaths than expected based on trends during the preceding 10 years (8).

Between 2000-2015, the CVD mortality gap between the U.S. and its peer countries widened (9). In the U.S., life expectancy declined between 2015-2017 due mainly to an increase in death rates in working-age adults (25-64 years) from stress-related causes: drug poisoning; alcohol-induced causes; suicide; and diabetes, obesity and CVD (9). Since 2000 deaths with hypertension as the underlying cause have also been increasing in the U.S. (10).

A National Academy of Sciences report indicated that “social, economic, and cultural changes that have undermined economic security, intergenerational mobility, and social support networks can adversely affect cardiometabolic health through stress-mediated biological pathways and reduced access to care” (9, p. e2). Labor market and socioeconomic factors, which shape work organization and psychosocial job hazards, have been worsening in the U.S. For example, annual work hours increased 14% between 1982-2016 from 1652 to 1883 (11). Job stressors, such as low job control, job strain, and work-family conflict, increased in the U.S. between 2002-2014 (12).

Differences between countries in working conditions and health may be influenced by differences in union density (13), Psychosocial Safety Climate ("the extent of management concern for worker psychological health") (13) and legislation (14). Do such factors help to explain worsening health status (including CVD and hypertension) in the U.S. compared to peer countries?

4. Need to broaden scope of interventions to be evaluated

Important organizational interventions, such as collective bargaining, worker cooperatives, or legislative and regulatory-level interventions, are rarely evaluated and thus are typically not included in intervention review articles. U.S. examples include laws providing for better nurse-patient staffing ratios (15), bans on mandatory overtime, paid sick days, paid family leave or retail worker schedule predictability (16). In Quebec, Canada, the LMRSST (law modernizing the occupational health and safety system) will oblige all employers to measure and analyze psychosocial risks at work (prioritizing workplace violence) starting in 2025: "Employers must now ensure that work-related psychosocial risks that may have an impact on workers' health are included in their prevention program or action plan" (17). Such interventions need to be evaluated for their impacts on BP and CVD.

5. Changes in psychosocial working conditions in the Quebec, Canada workplace ABP intervention study

In the Quebec, Canada workplace ABP intervention study, at 30-month follow-up, BP and hypertension significantly decreased in the intervention group, with no change in the control group. In addition, the researchers documented that, at 30-month follow-up, levels of three job

stressors had decreased in the intervention group: high job demands decreased from 50.1% to 45.4%, low co-worker support dropped from 53.9% to 48.9%, and low respect and esteem, decreased from 36.1% to 30.9%. However, no changes were seen in other job stressors, such as low job control, low supervisor support and low reward (19).

6. History of research on work stressors, ambulatory blood pressure and risk of hypertension

The impact of working conditions, especially work organization and psychosocial stressors, on ambulatory blood pressure and risk of hypertension, has been studied for the past 40 years. Research began at Cornell Medical Center-New York Hospital in New York City (20, 21) and has been expanded upon by research groups in a number of countries. A number of review papers have been published on this important research (22-25).

References:

1. Härmä M, Kecklund G, Tucker P. Working hours and health - key research topics in the past and future. Scand J Work Environ Health. 2024;50(4):233-243. doi:10.5271/sjweh.4157
2. Li J, Pega F, Ujita Y, Brisson C, Clays E, Descatha A, Ferrario MM, Godderis L, Iavicoli S, Landsbergis PA, Metzendorf MI, Morgan RL, Pachito DV, Pikhart H, Richter B, Roncaioli M, Rugulies R, Schnall PL, Sembajwe G, . . . Siegrist J. (2020). The effect of exposure to long working hours on ischaemic heart disease: A systematic review and meta-analysis from the WHO/ILO Joint Estimates of the Work-related Burden of Disease

and Injury. Environment International, 142, 105739. https://doi.org/10.1016/j.envint.2020.105739

3. Descatha A, Sembajwe G, Pega F, et al. The effect of exposure to long working hours on stroke: A systematic review and meta-analysis from the WHO/ILO Joint Estimates of the Work-related Burden of Disease and Injury. Environ Int. 2020;142:105746. doi:10.1016/j.envint.2020.105746
4. Baron RM, Kenny DA. The moderator-mediator variable distinction in social psychological research: conceptual, strategic, and statistical considerations. J Pers Soc Psychol. 1986;51(6):1173-1182.
5. Fadel M, Sembajwe G, Li J, et al. Association between prolonged exposure to long working hours and stroke subtypes in the CONSTANCES cohort. Occup Environ Med. 2023;80(4):196-201. doi:10.1136/oemed-2022-108656
6. Berkman LF, Kelly EL, Hammer LB, Mierzwa F, Bodner T, McNamara T, Koga HK, Lee S, Marino M, Klein LC, et al. Employee Cardiometabolic Risk Following a Cluster-Randomized Workplace Intervention From the Work, Family and Health Network, 2009–2013. American Journal of Public Health. 2023;113 (12) :1322-1331.
7. Lopez AD, Adair T. Is the long-term decline in cardiovascular-disease mortality in high-income countries over? Evidence from national vital statistics. Int J Epidemiol. 2019;48(6):1815-1823.
8. Woodruff RC, Tong X, Khan SS, et al. Trends in Cardiovascular Disease Mortality Rates and Excess Deaths, 2010-2022. Am J Prev Med. 2024;66(4):582-589.

9. Harris KM, Woolf SH, Gaskin DJ. High and Rising Working-Age Mortality in the US: A Report From the National Academies of Sciences, Engineering, and Medicine. JAMA. 2021;325(20):2045-2046.
10. Shah NS, Lloyd-Jones DM, O'Flaherty M, et al. Trends in Cardiometabolic Mortality in the United States, 1999-2017. JAMA. 2019;322(8):780-782.
11. Economic Policy Institute. Annual wages and work hours. Washington, DC: Economic Policy Institute;2019.
12. Myers S, Govindarajulu U, Joseph M, Landsbergis P. Changes in work characteristics over 12 years: Findings from the 2002-2014 US National NIOSH Quality of Work Life Surveys. Am J Ind Med. 2019;62(6):511-522.
13. Dollard MF, Neser D. Worker health is good for the economy: Union density and psychosocial safety climate as determinants of country differences in worker health and productivity in 31 European countries. Social Science & Medicine. 2013;92:114-123.
14. Niedhammer I, Sultan-Taïeb H, Parent-Thirion A, Chastang J-F. Update of the fractions of cardiovascular diseases and mental disorders attributable to psychosocial work factors in Europe. International archives of occupational and environmental health. 2021:1-15.
15. Aiken L, Sloane D, Cimiotti J, et al. Implications of the California Nurse Staffing Mandate for Other States. Health Services Research. 2010;45(4).
16. Healthy Work Campaign. Healthy Work Strategies. 2024; https://healthywork.org/resources/healthy-work-strategies/. Accessed June 30, 2024.
17. Commission des normes, de l'équité, de la santé et de la sécurité du travail (CNESST). Loi modernisant le régime de santé et de sécurité au travail /An Act to modernize the occupational health and safety system in brief. Search for « psychosocial risks ».

Accessed June 30, 2024.
https://www.cnesst.gouv.qc.ca/fr/organisation/documentation/lois-reglements/modernisation-sst.

18. Goldberg M, Carton M, Descatha A, Leclerc A, Roquelaure Y, Santin G, Zins M; CONSTANCES team. CONSTANCES: a general prospective population-based cohort for occupational and environmental epidemiology: cohort profile. Occup Environ Med. 2017 Jan;74(1):66-71. doi: 10.1136/oemed-2016-103678
19. Gilbert-Ouimet M, Vézina M, Brisson C, et al. Intervention study on psychosocial work factors and mental health and musculoskeletal outcomes. HealthCare Papers. 2011;Special issue 11:49-68.
20. Schnall PL, Pieper C, Schwartz JE, et al. The relationship between 'job strain,' workplace diastolic blood pressure, and left ventricular mass index. Results of a case-control study. JAMA 1990;263(14):1929-1935.
21. Schnall PL, Schwartz JE, Landsbergis PA, Warren K, Pickering TG. A longitudinal study of job strain and ambulatory blood pressure: results from a three-year follow-up. Psychosom Med. 1998;60(6):697-706.
22. Landsbergis P, Garcia-Rivas J, Juárez-García A, Choi B, Dobson M, Gomez V, et al. Occupational Psychosocial Factors and Cardiovascular Disease. In: Tetrick L, Fisher G, Ford M, Quick J, editors. Handbook of Occupational Health Psychology. 3. Washington, DC: American Psychological Association; 2024. p. 309-39.
23. Landsbergis P, Dobson M, Koutsouras G, Schnall P. Job strain and ambulatory blood pressure: A meta-analysis and systematic review. American Journal of Public Health. 2013;103(3):e61-e71.

24. Gilbert-Ouimet M, Trudel X, Brisson C, Milot A, Vezina M. Adverse effects of psychosocial work factors on blood pressure: systematic review of studies on demand-control-support and effort-reward imbalance models. Scand J Work Environ Health. 2014;40(2):109-32.
25. Trudel X, Brisson C, Gilbert-Ouimet M, Milot A. Psychosocial Stressors at Work and Ambulatory Blood Pressure. Curr Cardiol Rep. 2018;20(12):127.
